# Addressing a weakness of anticancer therapy with mitosis inhibitors: Mitotic slippage

**DOI:** 10.1080/23723556.2016.1277293

**Published:** 2017-01-05

**Authors:** Riju S. Balachandran, Edward T. Kipreos

**Affiliations:** Department of Cellular Biology, University of Georgia, Athens, GA, USA

**Keywords:** APC/C, antimicrotubule drugs, antimitotic chemotherapy, cullin-RING ubiquitin ligase, cyclin B1, mitotic slippage, tetraploid checkpoint, tetraploidy, ZYG11A, ZYG11B

## Abstract

Mitosis inhibitors, which include antimicrotubule drugs, are chemotherapy agents that induce the arrest and apoptosis of mitotic cells. Mitotic slippage, in which mitotically arrested cells exit mitosis, limits the effectiveness of mitosis inhibitors. We have discovered that the CRL2^ZYG11A/B^ ubiquitin ligase promotes mitotic slippage. The combination of antimicrotubule drugs and a CRL2^ZYG11A/B^ inhibitor prevents mitotic slippage to increase antimitotic efficacy.

Mitosis inhibitors (also known as antimitotic drugs) are chemotherapy agents that target cells in mitosis. Antimicrotubule drugs (also known as microtubule poisons or spindle poisons) are the most effective class of mitosis inhibitors.^1^ Antimicrotubule drugs act by either blocking the polymerization or depolymerization of microtubules.^2^ These drugs generally bind to three sites on microtubules: the vinca domain; colchicine domain; or taxane site. Drugs that target the vinca or colchicine domains (like vinblastine, vincristine, and colchicine) lead to the depolymerization of microtubules by preventing the addition of new tubulin subunits. Drugs that target the taxane site (like paclitaxel/taxol) stabilize microtubules by inhibiting depolymerization.

Antimicrotubule drugs target dividing cells by affecting the assembly or function of the mitotic spindle. Spindle microtubule dynamics are required for the alignment and segregation of mitotic chromosomes. Antimicrotubule drugs inhibit microtubule dynamics, leading to a failure to align the chromosomes at the metaphase plate and the activation of the spindle assembly checkpoint (SAC).^2^ Other categories of antimitotic drugs also function by activating the SAC.^1^ A constitutively active SAC arrests cells in mitosis, and many of the arrested cells undergo apoptosis.^2^

The mitotic arrest induced by antimicrotubule drugs is countered by a process called “mitotic slippage”.^2^ Mitotic slippage occurs when the cells arrested by the SAC exit mitosis without dividing. Mitotic slippage occurs *in vitro* in a substantial portion of cells with a constitutively activated SAC.^2^ When analyzed in mice, mitotic slippage has been found to occur at a much higher rate *in vivo* than *in vitro*.^3^

When cells undergo mitotic slippage, they exit mitosis without undergoing cytokinesis, and become tetraploid. Tetraploidy is an unwelcomed side effect of antimicrotubule drugs, as tetraploidy leads to aneuploidy and chromosomal translocations that promote cancer.^4^

After mitotic slippage, tetraploid cells have three potential fates: re-enter the cell cycle and undergo further rounds of cell division; enter a G1-phase cell cycle arrest; or undergo apoptosis. The tetraploid checkpoint can mediate the latter 2 fates. The tetraploid checkpoint functions through the activation of the Hippo pathway to inhibit the degradation of tumor protein p53 (TP53, best known as p53).^5^ Upon stabilization, p53 induces cell cycle arrest or apoptosis.

Notably, the tetraploid checkpoint requires p53, yet most solid tumors lack functional p53.^6^ Therefore, most cancer cells are unable to activate the tetraploid checkpoint. However, cells lacking functional p53 also die after mitotic slippage, suggesting the existence of alternative pathways that eliminate cells after mitotic slippage.^7^

When comparing different cell lines treated with antimicrotubule drugs, the occurrence of mitotic slippage and the outcomes associated with mitotic slippage vary substantially.^8^ However, even in cell lines in which the majority of cells die after mitotic slippage, there generally remains a percentage of cells that do not die and instead re-enter the cell cycle.^8^ The efficacy of antimicrotubule drug treatments could thus be substantially improved by ensuring that all cells die during the mitotic arrest, rather than relying on death after mitotic slippage.

The primary mechanism for mitotic slippage is the continued degradation of cyclin B1 (CCNB1) during the metaphase arrest.^9^ Cyclin dependent kinase 1 (CDK1) forms a complex with cyclin B1 that phosphorylates substrates to drive cells into mitosis. The cyclin B1–CDK1 complex must be inactivated through the degradation of cyclin B1 to allow cells to exit mitosis. The anaphase-promoting complex/cyclosome (APC/C) ubiquitin ligase is known to polyubiquitylate cyclin B1 at the metaphase-to-anaphase transition to target its degradation by the proteasome. During a normal mitosis, the APC/C is inhibited by the SAC until the chromosomes are properly aligned at the metaphase plate.

It has been shown that in cells with constitutively activated SAC, cyclin B1 degradation continues even in the absence of APC/C activity.^9^ We recently discovered that another ubiquitin ligase targets the degradation of cyclin B1 during SAC arrest to promote mitotic slippage: cullin-RING ubiquitin ligase 2 complexes that contain the zyg-11 family members A or B (ZYG11A or ZYG11B) as the substrate receptor; denoted CRL2^ZYG11A/B^.^10^

We found that CRL2^ZYG11A/B^ binds cyclin B1 and catalyzes its polyubiquitylation, which targets cyclin B1 for degradation by the proteasome (Fig. 1).^10^ The degradation of cyclin B1 by CRL2^ZYG11A/B^ is independent of APC/C, and the two ubiquitin ligases target different regions of the cyclin B1 protein to mediate the degradation. Under normal conditions, knockdown of ZYG11A/B does not appreciably affect cyclin B1 levels or mitotic progression. However, if APC/C is inhibited or cyclin B1 is overexpressed, ZYG11A/B becomes important for the metaphase-to-anaphase transition, and ZYG11A/B knockdown induces a prolonged metaphase arrest.^10^ The mitotic arrest appears to result from the continued presence of cyclin B1, as cells with both ZYG11A/B and APC/C inactivated will rapidly exit mitosis if treated with an inhibitor of cyclin B1–CDK1 activity.^10^

**Figure 1.**
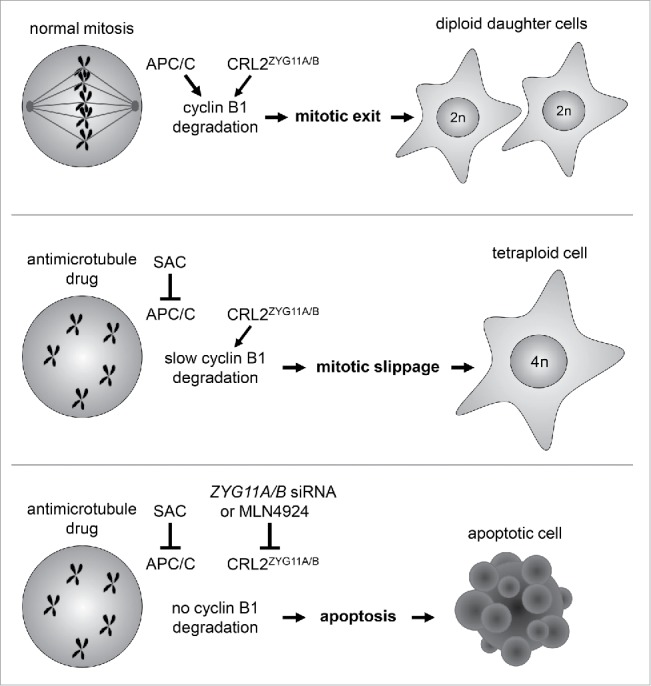
The regulation of mitotic slippage in cells treated with microtubule/spindle poisons. The top panel shows a cell undergoing normal mitosis. Both APC/C and CRL2^ZYG11A/B^ ubiquitin ligases target the degradation of the mitotic regulator cyclin B1 to allow cells to progress from metaphase to anaphase and undergo mitotic exit. The middle panel shows a cell treated with an antimicrotubule drug that depolymerizes microtubules. The unattached kinetochores on the chromosomes activate the SAC, which inhibits APC/C. The slow degradation of cyclin B1 mediated by CRL2^ZYG11A/B^ allows the cell to exit mitosis via mitotic slippage (after an arrest period). The bottom panel shows a cell treated with both an antimicrotubule drug and an inhibitor of CRL2^ZYG11A/B^ (*ZYG11A/B* small interfering RNA, siRNA, or the CRL inhibitor MLN4924). Cyclin B1 is not degraded and the cell dies by apoptosis during the mitotic arrest.

When the antimicrotubule drug nocodazole is added to cells, the SAC becomes constitutively activated, which then inactivates APC/C. The treatment of nocodazole-arrested cells with an APC/C inhibitor did not significantly reduce the level of mitotic slippage, suggesting that APC/C was already functionally inhibited and did not contribute to mitotic slippage in the SAC-arrested cells.^10^ In contrast, knockdown of ZYG11A/B in nocodazole-treated cells significantly reduced the occurrence of mitotic slippage.^10^ This indicates that CRL2^ZYG11A/B^ promotes mitotic slippage in SAC-arrested cells (Fig. 1).

Combination therapy is often used to increase the efficacy of cancer treatments. We observed that the combination of the antimicrotubule drug nocodazole and a small molecule inhibitor of all CRL ubiquitin ligases, MLN4924 (pevonedistat), completely abolished the occurrence of mitotic slippage in U2OS cells—leading to 100% cell death during the mitotic arrest.^10^ By itself, MLN4924 treatment did not affect the mitotic progression of U2OS cells.^10^

MLN4924 is currently undergoing phase 2 clinical trials as a cancer treatment. Our results suggest that the combination of MLN4924 with antimicrotubule drugs may increase the efficacy of antimicrotubule chemotherapy. However, because MLN4924 inhibits diverse CRL ubiquitin ligase complexes, it may be worthwhile to pursue more specific small molecule inhibitors of CRL2^ZYG11A/B^. Specifically targeting the CRL that promotes mitotic slippage may engender lower side effects, which could allow higher effective doses.
